# P-998. A multi-center observational study in Northern Italy to evaluate the impact of Sepsis bundle in Obstetric Settings: the SOS study

**DOI:** 10.1093/ofid/ofae631.1188

**Published:** 2025-01-29

**Authors:** Marta Colaneri, Simona Biscarini, Lara Tiranini, Rebecca Pesare, Pietro Valsecchi, Elena Maria Seminari, Arsenio Spinillo, Alessandra Bandera, Enrico Maria Ferrazzi, Andrea Gori, Laura Carenzi, Luigi Pusterla, Federico D’Amico, Alessandro Raimondi, Massimo Puoti, Elisa Vallicella, Gianpaolo Grisolia, Alice Zavatta, Irene Cetin, Alice Bonetti, Fausto Baldanti, Patrizia Cambieri, Angelo Pan, Elena Maria Seminari, Camilla Torriani, Cristina Maria Monti, Raffaele Bruno

**Affiliations:** Department of Biomedical and Clinical Sciences, University of Milan, Milan, Italy, Milano, Lombardia, Italy; IRCCS Policlinico Maggiore of Milan, MIlano, Lombardia, Italy; IRCCS Policlinico San Matteo, Pavia, Lombardia, Italy; Infectious Diseases Unit, IRCCS Policlinico San Matteo Foundation, Pavia, Italy, Pavia, Lombardia, Italy; Infectious Diseases Unit, IRCCS Policlinico San Matteo Foundation, Pavia, Italy, Pavia, Lombardia, Italy; Infectious Diseases Unit, IRCCS Policlinico San Matteo Foundation, Pavia, Italy, Pavia, Lombardia, Italy; IRCCS Policlinico San Matteo, Pavia, Lombardia, Italy; Department of Medical-Surgical and Transplant Pathophysiology, Fondazione IRCCS Ca’ Granda Ospedale Maggiore Policlinico, Milan, Italy, Milano, Lombardia, Italy; University of Milan, Milano, Lombardia, Italy; Infectious Diseases and Immunopathology, Department of Clinical Sciences, Università di Milano, Luigi Sacco Hospital, Milan, Italy, Milano, Lombardia, Italy; Infectious Diseases Unit, ASST Lariana ospedale sant'Anna, Como, Italy, Como, Lombardia, Italy; Infectious Diseases Unit, ASST Lariana ospedale sant'Anna, Como, Italy, Como, Lombardia, Italy; Ospedale Niguarda, Milan, Lombardia, Italy; Ospedale Niguarda, Milan, Lombardia, Italy; Department of Infectious Diseases, ASST Grande Ospedale Metropolitano Niguarda, Milan, Italy, Milano, Lombardia, Italy; Department of Obstetrics and Gynecology, ASST Ospedale Carlo Poma, Mantova, Italy, Mantova, Lombardia, Italy; Department of Obstetrics and Gynecology, ASST Ospedale Carlo Poma, Mantova, Italy, Mantova, Lombardia, Italy; Ospedale Buzzi, Milano, Lombardia, Italy; Department of Obstetrics and Gynecology, Fondazione IRCCS Ca' Granda Ospedale Maggiore Policlinico, Milan Italy, Milano, Lombardia, Italy; IRCCS Policlinico San Matteo, Pavia, Lombardia, Italy; IRCCS Policlinico San Matteo, Pavia, Lombardia, Italy; IRCCS Policlinico San Matteo, Pavia, Lombardia, Italy; Ospedale di Cremona, Cremona, Lombardia, Italy; IRCCS Policlinico San Matteo, Pavia, Lombardia, Italy; University of Pavia, Pavia, Lombardia, Italy; University of Pavia, Pavia, Lombardia, Italy; University of Pavia, Pavia, Lombardia, Italy

## Abstract

**Background:**

Maternal sepsis presents challenges in early detection and treatment. A pregnancy-specific sepsis scoring system (MEOWS) and management bundles for pregnant septic patients were recently introduced, but there is a lack of data on their effectiveness. Our study aims to assess the impact of implementing a Lombardy sepsis bundle for septic pregnant and puerperal patients.

**Table 1:**
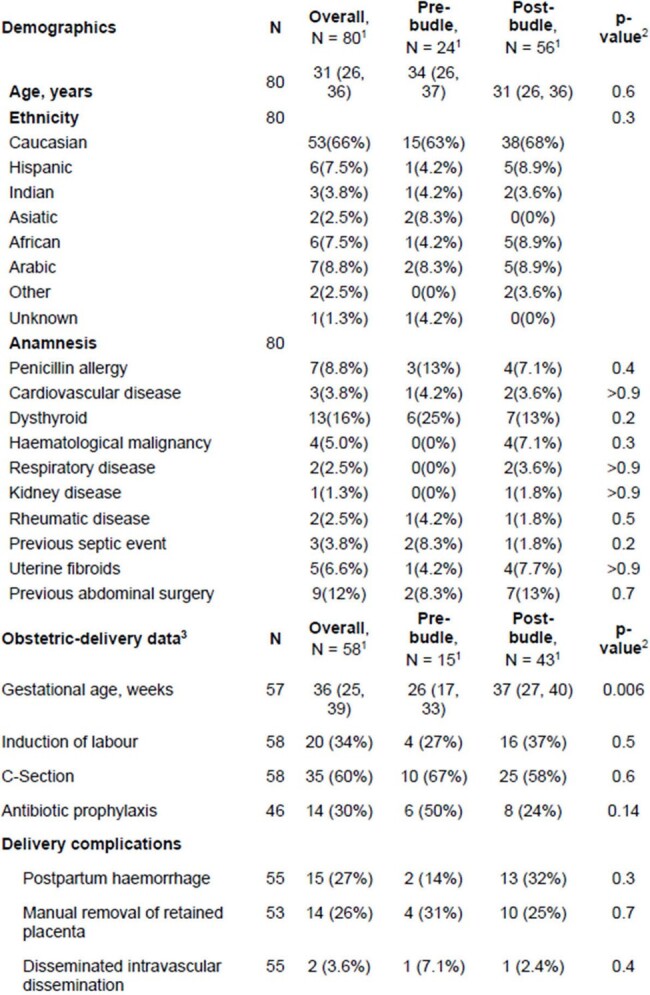
Demographic and clinical data of the patients included

**Methods:**

The SOS, a retrospective multicenter study across 7 Lombardy Hospitals, focuses on adult pregnant and puerperal women diagnosed with sepsis according to SCC [1]. Medical records from 2 periods were analyzed: pre-bundle implementation (May 2015 - May 2018) and post-bundle implementation (Jan 2019 - Jan 2022). The primary outcome was to describe clinical and microbiological data, while the secondary outcome was to evaluate the impact of the Sepsis bundle on the length of stay (LOS), the timing of antibiotic treatment, and the rate of positive blood cultures (BCs).

**Figure:**
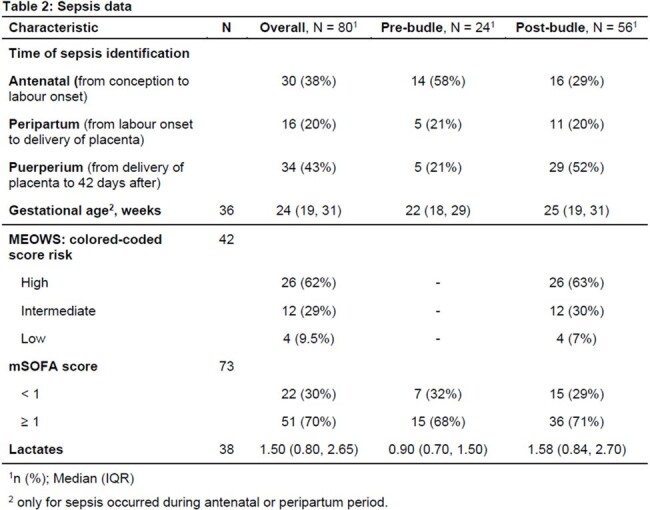

**Results:**

Eighty patients (24 in the pre-bundle and 56 in the post bundle period) were found (Table 1). Overall, median age was 31 years, they had few underlying comorbidities and mostly were pregnant (rather than puerperal) in the moment of sepsis occurrence (Table 2).

More than half of the post-bundle patients had a high MEOWS score (62%) and an mSOFA score of more than 1 (70%) (tablee 2). Microbiological data are detailed in Table 3, while outcome data in Table 4. No significant differences were found in the rate of positive BCs (p=1) between the 2 periods. Similarly, the rate of antibiotic treatment started before BCs collection, and the LOS did not significantly differ (p=0.40). However, in the post-bundle period there were both a marked increase in infectious disease (ID) consultations (p=0.003), and a reduction of neonatal Intensive Care Unit admission (p=0.013).

**Figure:**
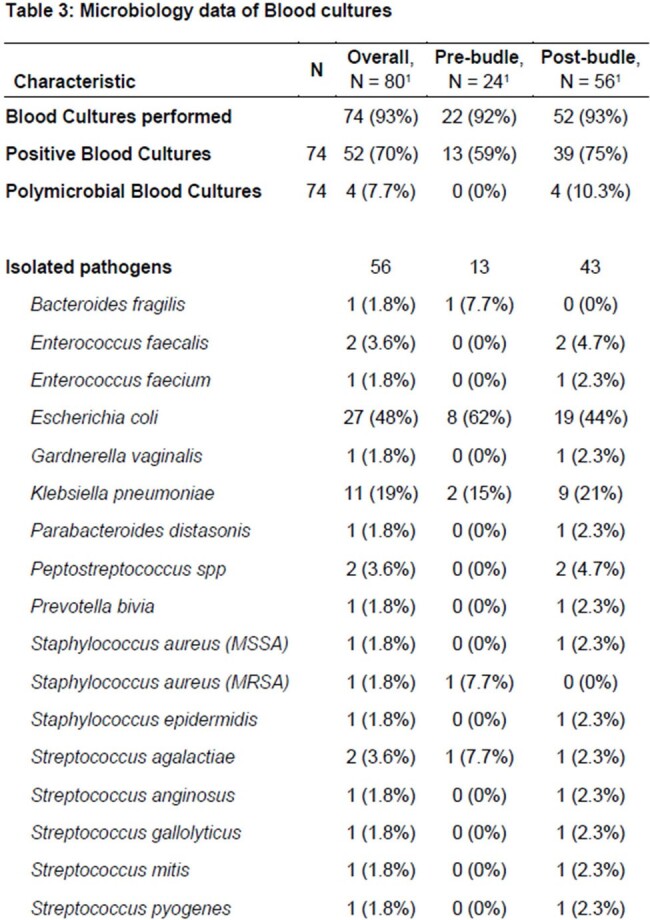

**Conclusion:**

While the bundle did not significantly improve maternal outcomes, it led to an increased number of ID consultations, providing a more comprehensive understanding of sepsis severity, and a consequent reduction of neonatal severity conditions.

**Figure:**
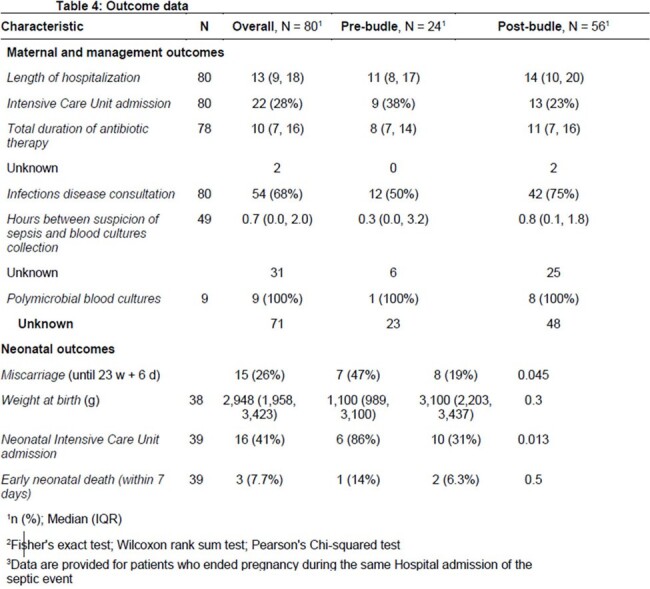

**Disclosures:**

**All Authors**: No reported disclosures

